# Casein kinase 1 isoform 2 is essential for bloodstream form *Trypanosoma brucei*^☆^

**DOI:** 10.1016/j.molbiopara.2009.03.001

**Published:** 2009-08

**Authors:** Michael D. Urbaniak

**Affiliations:** Division of Biological Chemistry and Drug Discovery, College of Life Sciences, University of Dundee, Dundee DD1 5EH, UK

**Keywords:** LmCK1.2, *Leishmania major* casein kinase 1 isoform 2, TbCK1.2, *Trypanosoma brucei* casein kinase 1 isoform 2, TbCK1.1, *Trypanosoma brucei* casein kinase 1 isoform 1, RNAi, RNA interference, GST, glutathione-S-transferase, PKs, protein kinases, *T. brucei*, *Trypanosoma brucei*, *L. Major*, *Leishmania major*, RT-PCR, reverse transcriptase-polymerase chain reaction, *Trypanosoma brucei*, Protein kinase, Genetic validation, RNA interference, Drug target

## Abstract

Induction of RNA interference targeted against casein kinase 1 isoform 2 (TbCK1.2, Tb927.5.800) in bloodstream form *Trypanosoma brucei in vitro* results in rapid cessation of growth, gross morphological changes, multinucleation and ultimately cell death. A null mutant of the highly homologous casein kinase 1 isoform 1 (Tb927.5.790) in bloodstream form *T. brucei* displays no growth or morphological phenotype *in vitro*. A truncated form of TbCK1.2 expressed in *Escherichia coli* as a GST fusion produces catalytically active recombinant protein, facilitating screening for small molecule inhibitors. These data show that TbCK1.2 is an attractive target for anti-trypanosomal drug discovery.

*Trypanosoma brucei* is the causative agent of African sleeping sickness in humans, and current treatments are expensive, toxic, and difficult to administer, leaving an urgent need for new therapeutic agents [1]. Drug discovery programs for African sleeping sickness have recently started in academia, but there remains a need to identify, validate and characterise new drug targets to feed this effort [2]. Protein kinases (PKs), key mediators of growth and cell signalling, are one of the major drug target families being tackled by the pharmaceutical industry. The *T. brucei* genome encodes 176 putative PKs [3], which are in theory attractive targets for anti-trypanosomal drug discovery, given the possibility of ‘piggy-back’ drug discovery [4].

The serine/threonine protein kinase casein kinase I family (CK1s) plays an important role in eukaryotic signalling pathways, and their substrates include key regulatory protein involved in cell differentiation, proliferation, chromosome segregation and circadian rhythms [5,6]. Essential CK1s are attractive targets for anti-trypanosomal drug discovery as CK1s are monomeric, constitutively active and usually co-factor independent, simplifying assay development, and there are several high resolution structures of CK1s with ATP or inhibitors bound [7] to aid rational drug design.

The *Leishmania major* CK1 isoform 2 (LmCK1.2, LmjF35.1010) has been implicated as an essential enzyme through studies using immobilized or radiolabelled inhibitors [8,9]. The same compounds were also shown to be cytotoxic to *T. brucei*, implying that the homologous *T. brucei* enzyme (TbCK1.2) may also be essential. Here, we demonstrate that TbCK1.2 is an attractive drug target by establishing its essentiality for the survival of bloodstream form *T. brucei*, and demonstrating that functional recombinant protein can be obtained to facilitate screening for inhibitors.

Recent bioinformatic analysis of the trypanosomatid genomes for the presence of PKs identified four CK1s in *T. brucei*, seven in *T. cruzi* and six in *L. major* [3]. Analysis of the protein sequences [10,11] revealed that two of the *T. brucei* CK1 proteins are highly homologous to the putatively essential LmCK1.2 (LmjF35.1010), namely TbCK1.2 (Tb927.5.800, 76% identity) and TbCK1.1 (Tb927.5.790, 62% identity). The TbCK1.1 and TbCK1.2 proteins are 72% identical to each other and occur on adjacent ORFs, raising the possibility that they may be functionally redundant. However, TbCK1.2 contains an unusual QQQQQQQQQQ motif located close to the C-terminus that is not present in either TbCK1.1 or LmCK1.2.

In order to investigate the essentiality of TbCK1.1 for the survival of bloodstream form *T. brucei* the haploid *TbCK1.1* genes were replaced with drug resistance genes by homologous recombination. Approximately 500 bp of the 5′- and 3′-UTR sequences immediately adjacent to *TbCK1.1* were PCR amplified from genomic DNA using primers that allowed the two products to be knitted together in a second PCR to create a restriction enzyme site between the UTRs, allowing a drug resistance gene to be inserted [12]. The puromycin acetyltransferase (PAC) and hygromycin phosphotransferase (HYG) drug resistance genes were inserted between the UTRs and the resulting constructs used sequentially to replace both alleles of *TbCK1.1* generating a Δ*TbCK1.1::PAC*/Δ*TbCK1.1::HYG* double knockout (dKO) cell line. Reverse transcriptase-PCR (RT-PCR) confirmed the absence of *TbCK1.1* mRNA and revealed that the *TbCK1.2* mRNA level was not significantly upregulated in response to the loss of *TbCK1.1*. The *TbCK1.1* dKO cell line had normal morphology (not shown) and its growth was unaltered compare to the wild type (Fig. 1A), demonstrating that TbCK1.1 is not essential *in vitro*.

The essentiality of the *TbCK1.2* gene for the survival of bloodstream form *T. brucei* was initially examined using the same methodology as applied to *TbCK1.1*. Although gene replacement of a single allele of *TbCK1.2* with either PAC or HYG to generate Δ*TbCK1.2::PAC* or Δ*TbCK1.1::HYG* single knockout cell lines was successful, the replacement of the second allele failed. To confirm the essentiality of *TbCK1.2*, several tetracycline-inducible RNAi cell lines were produced using a p2T7^TABlue^ vector [13] containing a 320-bp insert composed of 80-bp of 5’-UTR and the first 240-bp of the *TbCK1.2* ORF. After induction of RNAi by addition of tetracycline, RT-PCR confirmed a significant reduction in *TbCK1.2* mRNA levels with only a marginal decrease in *TbCK1.1* mRNA (Fig. 1B, inset). Ablation of *TbCK1.2* mRNA produced rapid cessation of growth (Fig. 1B), gross morphological changes and multinucleation (Fig. 1C), and ultimately cell death. Occasionally, so-called ‘revertant’ cells were observed, where, after an initial period of cell death, the growth of the cultures resumed. In such cases, RT-PCR revealed that *TbCK1.2* mRNA level had returned to wild type level in the revertant cells (not shown), suggesting spontaneous loss of tetracycline control may have occurred [12].

Taken together, these data demonstrate that the highly homologous casein kinases TbCK1.1 and TbCK1.2 are non-redundant, and that only TbCK1.2 is essential for the bloodstream form of the parasite *in vivo*. Knockdown of TbCK1.2 rapidly produces “monster” cells with gross morphological changes and multinucleation that clearly result from loss of cell cycle control; however, this does not necessarily indicate a direct role for CK1 in cytokinesis, as this phenotype may be the result of indirect effects [14]. As CK1s are known to be involved in diverse cellular processes [5,6], detailed analysis of the morphological changes induced by TbCK1.2 knockdown is unlikely to give a clear picture of the underlying biology. Instead, identifying the proteins that are phosphorylated by TbCK1.2, and the effect of such phosphorylation, may offer the best approach to understanding the cellular roles of TbCK1.2.

Despite the high level of sequence homology between TbCK1.1 and TbCK1.2, they are clearly functionally non-redundant. Typically, CK1 family members differ mainly in their non-kinase N- and C-terminal extensions, which have been proposed to influence localization and modulate activity [5,6]. TbCK1.2 contains an unusual C-terminal QQQQQQQQQQ motif that is absent from both TbCK1.1 and LmCK1.2, and although a similar C-terminal Q-rich region is found in several *Trypanosoma cruzi* CK1s (QQEQKQQQQQQ) and human CK1α (QGQQAQ), nothing is known about its significance.

Full length LmCK1.2 has previously been expressed in *Escherichia coli* as an active recombinant protein [9], however attempts to express full length TbCK1.2 produced little soluble protein. A truncated form of TbCK1.2 was designed based on the crystal structure of *Schizosaccharomyces pombe* Cki1Δ298, a truncated and active CK1 where 148 amino acids have been removed from the C-terminus [7]. The truncation of TbCK1.2, termed TbCK1.2Δ298, eliminates the C-terminal 34 amino acids including the unusual QQQQQQQQQQ motif. Expression of TbCK1.2Δ298 as a C-terminal GST fusion in *E. coli* and purification *via* immobilised glutathione produced a 61 kDa soluble protein whose identity was confirmed by tryptic peptide mass fingerprinting.

An inactive ‘kinase-dead’ form of TbCK1.2Δ298 was created by using site directed mutagenesis to alter the active site aspartate 133 to alanine (D133A), and was expressed and purified under identical conditions to the wild type protein. The ability of TbCK1.2Δ298 and TbCK1.2Δ298-D133A to phosphorylate myelin basic protein, partially dephosphorylated α-casein, a CK1 peptide substrate (RRKDLHDDEEDEAMSITA) and a CK1 phosphopeptide substrate (KRRRALS(p)VASLPGL) was measured in a γ[^33^P]-ATP filter plate assay. No autophosphorylation was observed, and whilst TbCK1.2Δ298 phosphorylated every substrate apart from MBP, the D133A ‘kinase-dead’ mutant was inactive, confirming that the catalytic activity observed was due to TbCK1.2Δ298 and not a contaminant. Determination of the kinetic parameters for TbCK1.2Δ298 kinase activity revealed modest *K*_m(app.)_ and *V*_max(app.)_ compared to those reported for recombinant LmCK1.2 (Table 1), suggesting that the optimal substrates for TbCK1.2 have not yet been identified.

Autophosphorylation of the C-terminus of CK1δ and CK1ɛ has been shown to down regulate kinase activity *in vitro*, although such phosphorylation is efficiently removed *in vivo* to maintain activity [6]. A recent proteome wide analysis of phosphorylation sites in *T. brucei* identified TbCK1.2 as being phosphorylated at the S19 position, with no C-terminal phosphorylation observed [16]. We did not observe any autophosphorylation *in vitro* with TbCK1.2Δ298, although the recombinant protein does lack the native C-terminus. If the phosphorylation of TbCK1.2 occurring at S19 is not due to autophosphorylation, it suggests that TbCK1.2 is phosphorylated *in vivo* by an upstream kinase that might modulate its activity.

In summary, we have demonstrated that TbCK1.2 is an essential enzyme for the clinically relevant bloodstream form of *T. brucei*, and have been able to obtain active and assayable recombinant protein. Whilst there is still much to learn about the biological role of TbCK1.2, these findings make TbCK1.2 an attractive target for antiparasitic drug discovery, as high-throughput screening for PK inhibitors is well established and should be readily adapted to a new kinase.

## Figures and Tables

**Fig. 1:**
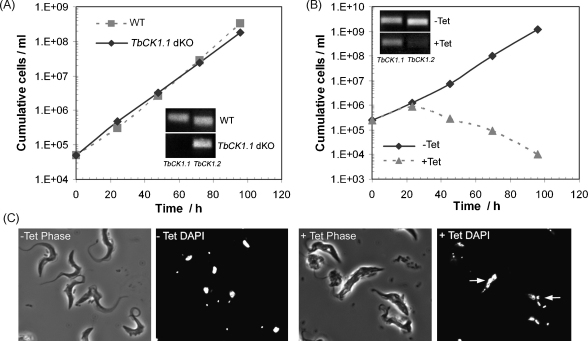
Growth and morphology of *TbCK1.1* knockout and *TbCK1.2* knockdown cells. (A) Growth of the *TbCK1.1* double knockout (dKO) cell line compared to wild type (WT), inset shows the RT-PCR analysis of *TbCK1.1* and *TbCK1.2* mRNA levels; (B) growth of *TbCK1.2* knockdown cells in the absence (−Tet) and presence (+Tet) of tetracycline, with RT-PCR inset; (C) Phase contrast and DAPI-stained microscopy of *TbCK1.2* knockdown cells cultured in the absence (−Tet) and presence (+Tet) of tetracycline for 48 h, arrows indicate multinucleation. The Δ*TbCK1.1::PAC*/Δ*TbCK1.1::HYG* dKO cell line was created by homologous recombination. Knockdown of *TbCK1.2* by tetracycline inducible RNAi was achieved with a *TbCK1.2* specific fragment [15] PCR-amplified from genomic DNA (primers 5′-GACAGCGGCAATAATCC-3′ and 5′-CCACAACACCGCCAC-3′) and cloned into p2T7^TAblue^ as described by Alibu et al. [13]. RT-PCR was performed using the Quick-Access RT-PCR system (Promega) using a common 5′-primer (5′-TGGCAGGGTTAAAGGC-3′) with two unique 3′-primers producing a 345 bp fragment for *TbCK1.1* (5′-GACGGGATGTTCATC-3′) and a 320 bp fragment for *TbCK1.2* (5′-TCGGTGTCATCACTC-3′). Microscopy was performed using cell fixed in 4% paraformaldehyde and stained with 2 μg/ml DAPI, with images acquired on a Zeiss Axiovert 200 M fluorescence microscope. Growth curves and microscopy images are representative examples of multiple experiments (*n* ≥ 3).

**Table 1:** Kinetic parameters for TbCK1.2Δ298 and LmCK1.2.

Substrate	TbCK1.2Δ298		LmCK1.2^a^	
	*K*_m(app.)_^b^	*V*_max(app.)_^b^	*K*_m(app.)_	*V*_max(app.)_
α-Casein	15 ± 2	1.0 ± 0.08	2	5
CK1 peptide	70 ± 10	1.9 ± 0.14	NA	NA
CK1 phosphopeptide	66 ± 5	2.3 ± 0.08	42	11

NA: not available. Recombinant GST tagged TbCK1.2Δ298 was obtained by expression from a pGEX-6P1 vector in *E. coli* BL21(DE3)pLysS (Novagen). Briefly, cells were induced with 250 μM IPTG for 18 h at 16 °C, resuspended in buffer A (50 mM Tris pH 8.0, 250 mM NaCl, 0.1% β-mercaptoethanol, 0.2 mM PMSF, 1 mM benzamidine) supplemented with 1% Triton TX100, 1 mM EDTA and 1 mM EGTA, and lysed by sonication. The GST tagged protein was bound to glutathione sepharose, washed extensively with wash buffer (buffer A, 0.03% Brij, 0.1 mM EGTA), eluted with wash buffer supplemented with 20 mM glutathione, dialysed against wash buffer supplemented with 10% glycerol (v/v), and stored at −80 °C. Phosphorylation was measured in a γ[^33^P]-ATP filter plate assay. Briefly, 25 mM HEPES pH 7.4, 16 mM MgCl_2_, 5 mM glycerophosphate, 1 mM DTT, 0.4 mg/ml BSA and 30 μM ATP was supplemented with 1.5 μCi γ[^33^P]–ATP, 50 nM kinase, and 0–112.5 μM substrate and the reaction allowed to proceed for 1 h. Reactions were stopped by addition of H_3_PO_4_, the phosphorylated substrates captured on a P81 filter plate (Whatman), washed three times with H_3_PO_4_, the plates dried, and the radioactivity counted on a Topcount NXT scintillation counter (PerkinElmer).

a Values for recombinant LmCK1.2 taken from Allocco et al. [9].

b Values for *K*_m(app.)_ (μM) and *V*_max(app.)_ (nmol/(min mg)) were calculated using non-linear regression analysis; the is data averaged from duplicate 8 point titrations and given ± standard error.
